# Production Performance of Four Pig Herds Infected With Porcine Reproductive and Respiratory Syndrome Using the “Load-Close-Exposure” Approach in China

**DOI:** 10.3389/fvets.2022.882971

**Published:** 2022-05-11

**Authors:** Zhendong Zhang, Xiangyang Qu, Xiaoquan Wang, Zhi Li, Shuqing Yang, Liumei Sun, Bin Zhou

**Affiliations:** ^1^School of Biotechnology, Jiangsu University of Science and Technology, Zhenjiang, China; ^2^Nanjing Dr. Vet Health Management CO., LTD, Nanjing, China; ^3^College of Veterinary Medicine, Nanjing Agricultural University, Nanjing, China

**Keywords:** PRRS control, intervention strategies, load-close-exposure, production performance, large-scale pig farms

## Abstract

Porcine reproductive and respiratory syndrome (PRRS) is one of the most important swine diseases causing enormous losses to pig producers all over the world. The intervention measure of “load-close-exposure” [interrupting the introduction of replacement pigs combined with whole-herd exposure to live PRRS virus (PRRSV)] has been widely used in North America and has shown wonderful outcomes in controlling PRRS in the field. In the present study, we performed analyses of the production performance of four herds acutely infected with PRRSV by adopting this measure for the first time in China. Our results showed that the development rate of gilts decreased by a mean of 8.56%, the farrowing rate of breeding sows decreased from 86.18 to 77.61%, the number of piglets born alive per sow decreased by a mean of 0.73 pigs, and the pre-weaning and post-weaning mortality of piglets increased by a mean of 2.74–4.97% compared to the parameters of 6 months before an outbreak. The time to PRRSV stability (TTS), defined as the time in weeks it took to produce PRRSV-negative pigs at weaning, is an important indicator of successful control of PRRSV. The median TTS among herds A, C, and D was 21.8 weeks (21.6 22.1 weeks). In herd B, TTS was 42.3 weeks, which could be explained by the double introduction of gilts. Our study suggests that the “load-close-exposure” strategy may be a good alternative for Chinese producers and veterinaries to control PRRS in the field.

## Introduction

Porcine reproductive and respiratory syndrome virus (PRRSV) is a small enveloped, positive single-stranded RNA virus belonging to the genus *Betaarterivirus* of the family *Arteriviridae* in the order *Nidovirales* (International Committee on Taxonomy of Viruses, ICTV)^1^, mainly leading to poor production performance and reproductive failure in sows and severe respiratory disorders in all age pigs (1). Since the first outbreak of PRRSV in the last century, it has spread rapidly and widely with a broad variation and extensive evolution, with almost all pig-producing countries being extremely affected by it (2, 3). A lot of studies have been conducted and explored to analyze the production and economic losses due to porcine reproductive and respiratory syndrome (PRRS) outbreaks (4, 5). In 2013, the combined production losses due to PRRS in the US pig industry were estimated to be as high as $663.91 million per year (6). China is the biggest pork-producing country and has the largest consumer market in the world. Especially, in recent years, it has witnessed a great and rapid development, with more than 441.6 million pigs at the end of 2017 (7). Therefore, it can be speculated that PRRSV can induce high and immeasurable losses for China in the long run.

Various strategies and lots of efforts have been developed and undertaken to combat and control PRRS in the field since the initial outbreak of PRRS in the last century. Vaccination has been the first choice for most pig producers as some positive effects have been observed after the use of modified live virus (MLV) (8). Eradication has proven nearly impossible due to the high transmissibility and persistence of PRRSV infection although some eradication trials were performed at regional and national levels (9–11). At the herd level, “avoiding the introduction of contaminated semen into the sow herd,” “gilt acclimation,” “vaccination,” and “management changes to reduce exposure to bacteria to eliminate losses, McRebel” (preventing the spread of pathogens in suckling pigs) are all useful strategies to produce PRRSV-negative (not infected) weaned piglets from sow herds (12). To eliminate PRRSV from sow herds, the three most popular methods are “test and removal,” “whole herd depopulation and repopulation,” and “load-close-exposure.” However, compared with the above mentioned two methods, “load-close-exposure” has been confirmed as the least expensive approach, being widely used to build protective immunity at the population level and to achieve the decrease and eventual elimination of PRRSV at herd and regional levels (13–15). This method consists of interrupting the introduction of incoming gilts into the breeding herd for at least 6 months (load-close) and whole-herd exposure to live PRRSV (exposure). Over the last few years, the “load-close-exposure” strategy has been introduced and adopted in practice in China, especially in large-scale pig production farms. However, no analysis on the efficiency and production impact of this strategy has been reported so far. In the present study, we acquired and analyzed the main production parameters in four PRRSV-infected pig farms after the “load-close-exposure” intervention, hoping to give a better understanding of the impact of PRRSV following a PRRS outbreak, providing some clues for the decision-making process to control PRRS in the field.

## Method

### Information About the Four Pig Farms and “Load-Close-Exposure” Strategy

Three pig farms (A, B, and C) with two-point production mode and one farrow-to-finish farm (D) of a large-scale swine company selected in the study consist of 5,100, 2,450, 3,800, and 1,750 sows, respectively. All four farms were in full-load production (about 5% of the sows were mated weekly) before a PRRS outbreak and were previously vaccinated with a MLV three times a year. TJM-F92, a kind of MLV derived from the highly pathogenic PRRSV strain TJ in China, were used in farm A, and Ingelvac PRRS^®^ MLV in the other three farms. The four farms were identified to be infected by PRRSV in November 2014, March 2015, December 2016, and February 2017, respectively, based on clinical signs discovered by veterinarians and laboratory diagnosis, with negative results for classical swine fever virus (CSFV), pseudorabies virus (PRV), and porcine circovirus 2 (PCV2). Four PRRSV strains (SDwh1403, SDqd1501, SDwh1601, and SDwh1701) were isolated from aborted fetuses collected from the four farms (16). Detailed information is shown in Supplementary Table 1.

At the time of a PRRS outbreak, all four farms adopted the “load-close-exposure” strategy. In farms A, C, and D, naive gilts (30–180 days of age) needed for replacement for the next 5–6 months were introduced at one time, but farm B performed an introduction two times at a 10-week interval due to insufficient availability of gilts. Gilts and other breeding sows were vaccinated two times with Ingelvac PRRS^®^ MLV at a 4-week interval in farms A and B. Gilts from farms C and D were first exposed to field live virus (FLV) and then vaccinated with Ingelvac PRRS^®^ MLV at a 4-week interval, and breeding sows were exposed to Ingelvac PRRS^®^ MLV two times (Supplementary Table 1). In all farrowing rooms, modified “McRebel” methods, such as cross-fostering only within 24 h, stop cross-fostering for weak piglets and the weak piglets should be executed immediately, all in-all out, and so on, were applied.

### Production Parameter Collection and Analysis

The main production parameters relevant to PRRS were acquired from the management system used in the farms, including: (1) culling rate, development rate for gilts; (2) mating rate after weaning (7 days), conception rate after mating (35 days), abortion rate per month, farrowing rate, piglets born alive per litter, stillbirths per litter, and mummies per litter for breeding sows; (3) weight at weaning (21 days), pre-weaning mortality, vaccine and medication costs for suckling piglets; and (4) mortality and culling rate, fattening days to reach 115 kg, feed conversion rate, vaccine and medication costs for nursery, and finishing pigs. For gilts, sows, and sucking piglets, we compared the production data between the first 6 months before a PRRS outbreak and the last 6 months after PRRS. For fattening pigs, the data acquired from the 6th to 10th month after a PRRS outbreak were compared to that of the 6 months before a PRRS outbreak, considering a growing time of 170 days (5–6 months) from birth (PRRSV positive) to slaughter.

### Serum Collection and Real-Time Quantitative PCR Detection

The time-to-stable (TTS) status for a PRRSV-infected herd is one of the important parameters to evaluate the efficiency of an intervention strategy. Twelve weeks after adopting the “load-close-exposure” strategy, serum was collected from weak pigs of weaning age (7 days before and 3 days after weaning; one pig per litter) to perform PRRSV detection using real-time quantitative PCR (qRT-PCR; in pools of five) every 2 weeks as described before (17). The herd was not defined as stable until four consecutive negative PCR results were obtained. The modified monitoring program of “four consecutive tests”: 30 samples were subjected to the first test if all samples were negative; a second test was performed with 60 samples if all were negative; 120 samples would be acquired for the third test; similarly, the final fourth test was performed with 120 samples.

## Results

### The Production Impact Due to a PRRS Outbreak

Two important variables were analyzed for introduced gilts. Culling rates for herds A, B, C, and D between 6 months before a PRRS outbreak were 3.84, 4.66, 4.46, and 4.97%, and increased to 7.10, 6.29, 8.74, and 8.51% after an outbreak, respectively, with an increase from 4.48 to 7.66%. The development rate of all four farms was above 90%, but was negatively affected to varying degrees after a PRRS outbreak, with a mean decrease of 8.56% (7.16–10.33%). In addition, to provide sufficient replacements, the pigs at 30–180 days of age were introduced into the program, but low age pigs (30–70 days) showed obvious clinical manifestation, especially when exposed to FLV. The development rate for herds C and D was only 69.08 and 70.58% for these low age pigs, suggesting that the age of the gilts introduced was important when using the FLV exposure program. For breeding herds, we analyzed seven production parameters that are mainly affected by PRRSV infection. The mating rate after weaning (7 days) was 88.35% before a PRRS outbreak, but decreased to 83.37%, with a decline of 4.98% on average, and the influence was mainly concentrated in the first 3 months and returned to basic performance by the 6th month, except in herd B (Supplementary Table 2). The conception rate after mating (35 days) decreased from 92.16 (90.42–94.41%) to 87.33% (86.83–92.27%), with an average decrease of 5.27%. The conception rate was only 25.60% in herd D at the 3rd month after PRRSV infection; however, there was no obvious change in herd C, suggesting a large variation among the different herds (Supplementary Table 3). The abortion rate was only 0.92% per month before PRRS infection and increased to 4.05% after infection. Compared to the other three herds, a persistently higher abortion rate was observed in herd A in all months (1st−6th) after an outbreak, with the highest abortion rate of 17.66% in the 2nd month (Supplementary Table 4). Farrowing rates for herds A, B, C, and D between 6 months before a PRRS outbreak were 89.51, 86.31, 83.16, and 85.73%, and decreased to 83.73, 81.00, 71.09, and 74.62% after an outbreak, respectively, with a decline from 86.18 to 77.61%. Herds C and D were the most affected farms, with a decrease of 12.08% and 11.12% in the farrowing rate. The affected stage was mainly between the 3rd and 6th month for herd A and between the 3rd to 5th month for herd D, but the farrowing rate of herd C was affected 6 months after a PRRS outbreak (Supplementary Table 5). The mean number of piglets born alive/litter was 11.47 (10.93–11.91), but decreased to 10.75 (10.46–10.94), with a loss of 0.72 pigs for PRRSV-affected sows, including an increased number of stillbirths (0.56 pigs) and mummies (0.17 pigs) per litter. The production parameters of breeding sows and gilts due to a PRRS outbreak are summarized in Table 1.

**Table 1:** The effects of a porcine reproductive and respiratory syndrome (PRRS) outbreak on the main production parameters of breeding sows and gilts^#^.

**Herd types**	**Productive parameters**	**Before a PRRS outbreak**					**After a PRRS outbreak**					**Difference (before/after a PRRS outbreak)**				
		**A**	**B**	**C**	**D**	**Mean**	**A**	**B**	**C**	**D**	**Mean**	**A**	**B**	**C**	**D**	**Mean**
Gilts	Cull rate (%)	3.84	4.66	4.46	4.97	**4.48**	7.10	6.29	8.74	8.51	**7.66**	+3.27	+1.63	+4.28	+3.54	**+3.18**
	Development rate (%)	91.48	92.44	90.68	90.39	**91.25**	82.98	85.28	82.45	80.06	**82.69**	−8.50	−7.16	−8.23	−10.33	–**8.56**
Breeding sows	Mating rate after weaning (7 days) (%)	84.75	94.53	84.87	89.25	**88.35**	80.35	85.20	81.85	86.07	**83.37**	−4.40	−9.33	−3.02	−3.18	–**4.98**
	Conception rate after mating (35days) (%)	94.41	90.42	91.84	93.77	**92.61**	91.61	86.83	92.27	78.62	**84.32**	+2.80	+3.59	+0.43	+15.15	**+5.28**
	Abortion rate per month (%)	0.92	0.69	0.63	1.44	**0.92**	7.56	2.18	3.70	2.77	**4.05**	+6.63	+1.49	+3.06	+1.33	**+3.13**
	Farrowing rate (%)	89.51	86.31	83.16	85.73	**86.18**	83.73	81.00	71.09	74.62	**77.61**	−5.78	−5.31	−12.08	−11.12	–**8.57**
	Stillbirths per litter (pig)	0.52	0.44	0.43	0.36	**0.44**	0.91	0.98	1.18	0.88	**0.99**	+0.39	+0.54	+0.75	+0.53	**+0.56**
	Mummies per litter (pig)	0.23	0.26	0.20	0.31	**0.25**	0.47	0.45	0.32	0.45	**0.42**	+0.24	+0.20	+0.12	+0.14	**+0.17**
	Piglets born alive per litter (pig)	10.93	11.58	11.47	11.91	**11.47**	10.46	10.94	10.64	10.93	**10.75**	−0.48	−0.65	−0.83	−0.98	–**0.72**

# *The “mean” of parameters was marked with bold*.

*The “+” means the increase, and the “–” means the decrease compared to the parameters before outbreak*.

Three variables, weight at weaning (21 days), pre-weaning mortality, and vaccination and medication costs, were analyzed for suckling piglets (Table 2). The mean pre-weaning mortality was 9.91% before infection but increased to 12.67% after infection, ranging from 10.42 to 17.08%. A greater impact (from 10.09 to 17.08%) was observed for herd B, and the pre-weaning mortality in the 1st and 2nd month was up to 32.85 and 21.06% with a gradual decline after 3 months (Supplementary Table 6). The weight at weaning (21 days) was negatively affected, decreasing from 6.50 to 6.03 kg. In addition, higher vaccination and medication costs were calculated and the mean increase per weaned piglet was ¥17.85 (from ¥20.42 to ¥38.275; ¥: the symbol of RMB). The main production parameters for 6 months before a PRRS outbreak and the 6th to 10th month after PRRSV infection were acquired to analyze the impacts of PRRS on nursery and finishing pigs (Table 3). An increase in mortality and culling rate in finishing pigs ranged from 2.85 to 9.58%, with an average of 4.98%. The mean number of fattening days to reach 115 kg after PRRSV infection increased to 181.36 days, with an average delay of 5.78 days, which represented a mean decrease of 3.29% for the average daily gain (ADG). A minimal impact (1.23 days) was founded for herd A and a large influence (10.05 days) for herd B from the herd level, and detailed information is given in Supplementary Table 7. In addition, the feed conversion rate increased from 2.59 to 2.68, with an average of 0.1 and vaccination and medication costs increased by more than ¥10 per finished pig in the outbreak period (Supplementary Table 8).

**Table 2:** The effects of a PRRS outbreak on the main production parameters of suckling piglets^#^.

	**Pre-weaning mortality (before/after, difference)**	**Weight at weaning (21 days) (before/after, difference)**	Health costs (¥)^*^(before/after, difference)
A	9.93%/10.61%, −0.68%	6.56/5.93, 0.63	20.62/36.37, −15.75
B	10.90%/17.08%, −6.18%	6.41/5.84, 0.57	22.37/41.98, −19.62
C	9.43%/12.57%, −3.14%	6.64/6.33, 0.31	19.08/37.93, −18.85
D	9.42%/10.42%, −1.00%	6.37/6.04, 0.33	19.62/36.80, −17.18
Mean	9.92%/12.67%, −2.75%	6.50/6.04, 0.46	20.42/38.27, −17.85

# *“+” means the increase, and the “–” means the decrease compared to the parameters before outbreak*.

* *Including vaccine and medication*.

**Table 3:** The effects of a PRRS outbreak on the main production parameters of nursery and finisher pigs^#^.

	**Mortality and cull rate (before/after, difference)**	**Fattening days to reach 115kg (before/after, difference)**	**Feed conversion rate (before/after, difference)**	Health costs (¥)^*^(before/after, difference)
A	7.37/10.22, −2.85	177.34/178.58, 1.23	2.58/2.67, 0.09	33.79/38.00, 4.21
B	5.63/15.21, −9.58	173.54/183.59, 10.05	2.61/2.73, 0.12	30.83/46.59, 15.77
C	9.17/12.99, −3.82	179.92/185.95, 6.03	2.59/2.69, 0.10	40.48/50.25, 9.78
D	6.13/9.78, −3.65	171.50/177.32, 5.82	2.57/2.65, 0.08	30.03/41.86, 11.83
Mean	7.07/12.05, −4.98	175.58/181.36, 5.78	2.59/2.69, 0.10	33.78/44.18, 10.40

# *“+” means the increase, and the “–” means the decrease compared to the parameters before an outbreak*.

* *Including vaccine and medication*.

### The Analysis of TTS

The “time to PRRSV stability” in pre-weaned pig herds is an important indicator to control PRRSV. A strict “30-60-120-120” modified monitoring program was enrolled to detect PRRSV in weaning-age pigs to determine the TTS. As shown in Table 4, consecutive PRRSV-negative results were observed in the 6th, 5th, and 5th test for herds A, C, and D, which represented TTS as 22.1, 21.7, and 21.6 weeks, respectively. However, it took a long period (42.3 weeks) to reach four consecutive negative results for herd B, which were probably attributable to the second loading at 10 weeks after PRRSV infection.

**Table 4:** The results of real-time quantitative PCR (qRT-PCR) for weaning piglets from different sow herds after 12 weeks of herd closure.

	**1st**		**2nd**		**3rd**		**4th**		**5th**		**6th**		**7th**		**8th**	
A	0/6	0%	0/6	0%	1/6	16.67%	1/12	8.33%	2/12	16.67%	0/12	0%	0/24	0%	0/24	0%
B	6/6	100%	0/6	0%	0/12	0%	2/12	16.67%	6/12	50%	4/12	33.33%	2/12	16.67%	5/12	41.67%
C	8/8	100%	6/6	100%	6/6	100%	2/6	33.33%	0/6	0%	0/12	0%	0/24	0%	0/24	0%
D	4/6	66.67%	3/6	50%	2/6	33.33%	1/6	16.67%	0/6	0%	0/12	0%	0/24	0%	0/24	0%

## Discussion

An optimized method combining introduction, herd closure, and MLV (load-close-exposure) was gradually popularized and performed in Chinese large-scale farms in recent years. In the present study, we firstly monitored and analyzed the production performance of four herds infected with PRRSV after using this method. The first step of the program was the introduction of replacement pigs into the breeding herd for at least 5–6 months. Strictly speaking, herd B should be dropped out because it underwent two introductions due to insufficient gilts being available at that time. Compared to the other three herds, culling and development rates were better for herd B, but the mating rate after weaning (7 days), pre-weaning mortality, mortality and culling rate of finishers were terrible, and even it took more than 20 weeks for herd B to produce PRRSV-negative piglets, which restresses the importance of performing once through gilt introduction and complete closure (decreasing the number of susceptible animals in which PRRSV can replicate and circulate) when controlling PRRS outbreaks. Linhares et al. (18) compared the effectiveness of two exposure programs (MLV and FLV) and observed that FLV herds achieve TTS faster than MLV herds, which might be due to the faster and stronger immune response induced by FLV. However, FLV was only used in the gilts introduced once in herds C and D because higher mortality was observed when the younger gilts were exposed to FLV, especially for piglets <70 days and no obvious difference was observed in TTS between MLV (herd A) and FLV (C and D) in the present study, further studies should be conducted to compare and find the most effective exposed measures.

In our present study, a total of 16 production parameters relevant to PRRSV were analyzed in detail. Four different PRRSV strains, SDwh1403 (a recombinant between NADC30-like and MLV), SDqd1501 (a recombinant between HP-PRRSV-like and QYYZ), SDwh1601 (a recombinant between JXA1-P80 (an MLV derived from HP-PRRSV) and NADC30-like), and SDwh1701 (a strain evolved from JXA1-P80) were isolated from herds A, B, C, and D, which might be the most important factor contributing to the different performance among the four herds (19–21). All four outbreaks occurred in breeding herds with an obviously increased abortion rate (Supplementary Table 4), and the development rate of gilts and farrowing rate of breeding sows were greatly influenced as a whole, both above 8%, suggesting that it was very important to return to baseline production to adopt proper strategies to stabilize gilts and breeding sows after PRRSV infection. In farrowing rooms, a modified “McReble” was strictly performed, so the pre-weaning mortality after infection was not significantly different from the data before a PRRS outbreak (Supplementary Table 6), except for the 1st and 2nd month in herd B (initial outbreak in the farrowing room and first infected with QYYZ-like strain), suggesting that the “McReble” is an effective method to control PRRS in piglets. For fattening pigs, mean health costs increased from ¥33.78 to ¥44.18, mainly including medicines used to control secondary bacterial infections. In herd B, a higher number of fattening days were observed to reach 115 kg (more than 10 days delay), which might be associated with the persistence of PRRSV infection in weaned piglets.

The breeding herd was classified as “positively stable” once PRRSV infection was controlled and ultimately eliminated based on previously proposed terminology, usually based on the confirmation of a sustained absence of detectable viremia in weaning-age pigs sampled for a minimum of 90 days (17). All four herds ultimately reached “positive stability” by using an optimized and strict monitoring program (“30-60-120-120”). The mean TTS was 21.8 weeks in herds A, C, and D, but it took 26.6 weeks on average in the infected 47 herds that recovered stable and were monitored by Linhares et al. (18); the difference of nearly 5 weeks might be attributed to a few “success” cases in our study. Linhares et al. (18) also reported that herds could achieve TTS sooner if the breeding sows had prior contact with PRRSV, all four herds were vaccinated three times a year, which might be another reason for the fewer weeks in our study. In addition, TTS was also found to be shorter in breeding herds that had natural exposure of gilts and sows to PRRSV (no deliberate exposure of PRRSV to sows or gilts) (22), implying that the resilience of pigs may also play a role in this, but it should be pointed out that the total loss (number of un-weaned pigs attributed to PRRS) was numerically lower for natural exposure (22). It took 42.3 weeks for the incompletely closed herd B (excluded from the analysis) to achieve stability, which restressed the importance of performing only one glit introduction followed by complete closure when controlling PRRS outbreaks.

In China, there exist a large number of pig farms with a diversity of size, different level of management and biosecurity. Many strategies are effective against PRRS, but not all of them have been proven to be the same in all cases. Thus, this study was only a quasi-experiment in which the metrics of “load-close-exposure” projects for many Chinese pig farms were analyzed. The strength of this study is the nature of the investigation all the four farms were from one company, and the acquired data were available and relatively accurate. However, the existence of unknown confounding variables might have biased the findings. Therefore, more precisely designed investigations, involving more herds, even with economic evaluation after different interventions, need to be studied in the future.

In conclusion, this quasi-experimental study explored performance data from four PRRSV-infected herds, suggesting that “load-close-exposure” is a choice to control PRRS in the field although productivity was also negatively affected. The key to return to baseline performance and achieving stability depends largely on introducing enough gilts at a time, closing the herd completely and performing a strict “McReble” in the farrowing room.

## Data Availability Statement

The original contributions presented in the study are included in the article/Supplementary Material, further inquiries can be directed to the corresponding author.

## Ethics Statement

Ethical review and approval was not required for the animal study because Blood sampling was done in the course of routine diagnostics on farm, therefore animal ethics committee approval was not required.

## Author Contributions

ZZ and XQ interpreted the data and drafted this manuscript. XQ coordinated the study. ZL and SY acquired the data from the management system used in the farms. XW and LS analyzed the data. BZ advised on the case study analysis. All authors read and approved the final manuscript.

## Funding

This work was supported by the Natural Science Foundation of Jiangsu Province (BK20201005) and Nanjing Dr. Vet Health Management CO., LTD.

## Conflict of Interest

XQ, ZL, and SY was employed by Nanjing Dr. Vet Health Management CO Limited. The remaining authors declare that the research was conducted in the absence of any commercial or financial relationships that could be construed as a potential conflict of interest.

## Publisher's Note

All claims expressed in this article are solely those of the authors and do not necessarily represent those of their affiliated organizations, or those of the publisher, the editors and the reviewers. Any product that may be evaluated in this article, or claim that may be made by its manufacturer, is not guaranteed or endorsed by the publisher.

## Footnotes

^1^ https://talk.ictvonline.org/taxonomy/p/taxonomy-history?taxnode_id=20171832
