# Supplementary material for: Production Performance of Four Pig Herds Infected With Porcine Reproductive and Respiratory Syndrome Using the “Load-Close-Exposure” Approach in China
Source: Front Vet Sci. 2022 May 11;9:882971. doi: 10.3389/fvets.2022.882971 (PMC9132538; doi:10.3389/fvets.2022.882971)
Supplement: Supplementary file 1 [file Data_Sheet_1.docx]

**Supplementary data**

**Table S1. The basic information of four porcine reproductive and respiratory syndrome virus (PRRSV) infected herds**

| Farm | Farm type | Herd size (sows) | Outbreak time | Vaccination strain^*^ | Isolated strain | Exposed virus | |
| --- | --- | --- | --- | --- | --- | --- | --- |
|  |  |  |  |  |  | gilts | breeding sows |
| A | two-point^#^ | 5100 | 2014.11 | TJM-F92 | SDwh1403 | Ingelvac+ Ingelvac | Ingelvac+ Ingelvac |
| B | two-point | 2450 | 2015.3 | Ingelvac | SDqd1501 |  |  |
| C | two-point | 3800 | 2016.12 | Ingelvac | SDwh1601 | FLV+ Ingelvac |  |
| D | farrow-to-finish | 1750 | 2017.2 | Ingelvac | SDwh1701 |  |  |

* TJM-F92 means a HP-PRRSV MLV strain, Ingelvac means IngelvacPRRS MLV.

^#^”two-point” means the breeding farms with sale of piglets at weaning.

**Table S2. The mating rate after weaning (7 days) per month**

| Farm | Mating rate after weaning (7 days) before outbreak (6 months) | | | | | | | Mating rate after weaning (7 days) after outbreak (6 months) | | | | | | | Difference |
| --- | --- | --- | --- | --- | --- | --- | --- | --- | --- | --- | --- | --- | --- | --- | --- |
|  | -6^th^ | -5^th^ | -4^th^ | -3^rd^ | -2^nd^ | -1^st^ | mean | +1^st^ | +2^nd^ | +3^rd^ | +4^th^ | +5^th^ | +6^th^ | mean |  |
| A | 81.92% | 90.56% | 83.06% | 86.12% | 83.55% | 83.29% | 84.75% | 77.57% | 72.19% | 77.02% | 81.56% | 84.96% | 88.77% | 80.35% | 4.41% |
| B | 98.85% | 90.59% | 92.58% | 96.68% | 93.99% | 94.47% | 94.53% | 90.80% | 88.10% | 87.04% | 83.28% | 82.20% | 79.79% | 85.20% | 9.32% |
| C | 87.08% | 89.90% | 80.92% | 82.15% | 84.90% | 84.28% | 84.87% | 77.66% | 82.50% | 86.53% | 83.42% | 76.40% | 84.58% | 81.85% | 3.02% |
| D | 84.80% | 84.80% | 88.00% | 91.10% | 91.50% | 95.30% | 89.25% | 80.50% | 87.60% | 85.4% | 82.8% | 88.9% | 91.2% | 86.07% | 3.18% |

**Table S3. The conception rate after mating (35 days) per month***

| Farm | Conception rate after mating (35 days) before outbreak (6 months) | | | | | | | Conception rate after mating (35 days) after outbreak (6 months) | | | | | | | Difference |
| --- | --- | --- | --- | --- | --- | --- | --- | --- | --- | --- | --- | --- | --- | --- | --- |
|  | -6^th^ | -5^th^ | -4^th^ | -3^rd^ | -2^nd^ | -1^st^ | mean | +1^st^ | +2^nd^ | +3^rd^ | +4^th^ | +5^th^ | +6^th^ | mean |  |
| A | 95.40% | 95.11% | 94.49% | 93.12% | 95.37% | 92.96% | 94.41% | 95.20% | 90.32% | 90.56% | 92.04% | 88.08% | 93.44% | 91.61% | -2.80% |
| B | 90.43% | 87.25% | 88.31% | 89.40% | 91.19% | 95.96% | 90.42% | 89.19% | 87.15% | 86.85% | 86.40% | 85.37% | 86.02% | 86.83% | -3.59% |
| C | 92.02% | 87.25% | 94.12% | 91.95% | 92.50% | 93.18% | 91.84% | 91.76% | 88.53% | 95.13% | 93.24% | 92.78% | 92.16% | 92.27% | 0.43% |
| D | 94.00% | 90.10% | 93.60% | 96.10% | 92.70% | 96.10% | 93.77% | 88.20% | 87.50% | 25.60% | 92.50% | 88.90% | 89.00% | 78.62% | -15.15% |

*****The conception was confirmed by B-ultrasound diagnosis.

**Table S4. The abortion rate of gestation sows per month**

| Farm | Abortion rate before outbreak (6 months) | | | | | | | Abortion rate after outbreak (6 months) | | | | | | | Difference |
| --- | --- | --- | --- | --- | --- | --- | --- | --- | --- | --- | --- | --- | --- | --- | --- |
|  | -6^th^ | -5^th^ | -4^th^ | -3^rd^ | -2^nd^ | -1^st^ | mean | +1^st^ | +2^nd^ | +3^rd^ | +4^th^ | +5^th^ | +6^th^ | mean |  |
| A | 0.52% | 0.72% | 1.10% | 0.68% | 1.32% | 1.20% | 0.92% | 4.25% | 17.66% | 8.24% | 5.29% | 5.86% | 4.03% | 7.56% | 6.63% |
| B | 0.45% | 0.89% | 0.67% | 0.35% | 0.60% | 1.16% | 0.69% | 3.36% | 1.41% | 1.65% | 2.07% | 2.09% | 2.48% | 2.18% | 1.49% |
| C | 0.51% | 0.68% | 0.73% | 0.45% | 0.61% | 0.82% | 0.63% | 12.27% | 3.45% | 1.31% | 2.12% | 1.42% | 1.61% | 3.70% | 3.06% |
| D | 1.86% | 1.13% | 0.97% | 0.94% | 1.71% | 2.03% | 1.44% | 5.23% | 5.60% | 2.16% | 1.41% | 0.89% | 1.34% | 2.77% | 1.33% |

**Table S5. The farrowing rate of gestation sows per month**

| Farm | Farrowing rate before outbreak (6 months) | | | | | | | Farrowing rate after outbreak (6 months) | | | | | | | Difference |
| --- | --- | --- | --- | --- | --- | --- | --- | --- | --- | --- | --- | --- | --- | --- | --- |
|  | -6^th^ | -5^th^ | -4^th^ | -3^rd^ | -2^nd^ | -1^st^ | mean | +1^st^ | +2^nd^ | +3^rd^ | +4^th^ | +5^th^ | +6^th^ | mean |  |
| A | 81.31% | 90.81% | 90.93% | 89.93% | 92.10% | 91.95% | 89.51% | 91.34% | 89.47% | 78.41% | 78.42% | 81.30% | 83.44% | 83.73% | -5.78% |
| B | 91.36% | 76.32% | 88.29% | 89.17% | 85.86% | 86.87% | 86.31% | 78.46% | 80.75% | 77.46% | 82.79% | 83.83% | 82.70% | 81.00% | -5.31% |
| C | 85.42% | 82.38% | 79.32% | 83.83% | 86.03% | 81.98% | 83.16% | 61.12% | 69.03% | 70.73% | 69.32% | 77.76% | 78.55% | 71.09% | -12.08% |
| D | 83.90% | 88.30% | 85.30% | 79.70% | 85.40% | 91.80% | 85.73% | 91.80% | 84.70% | 77.70% | 81.70% | 24.40% | 87.40% | 74.62% | -11.12% |

**Table S6. The pre-weaning mortality of suckling piglets per month**

| Farm | Farrowing rate before outbreak (6 months) | | | | | | | Farrowing rate after outbreak (6 months) | | | | | | | Difference |
| --- | --- | --- | --- | --- | --- | --- | --- | --- | --- | --- | --- | --- | --- | --- | --- |
|  | -6^th^ | -5^th^ | -4^th^ | -3^rd^ | -2^nd^ | -1^st^ | mean | +1^st^ | +2^nd^ | +3^rd^ | +4^th^ | +5^th^ | +6^th^ | mean |  |
| A | 9.28% | 8.73% | 10.54% | 9.30% | 11.43% | 10.27% | 9.93% | 12.88% | 11.41% | 9.72% | 8.79% | 10.11% | 10.73% | 10.61% | -0.68% |
| B | 11.97% | 10.43% | 9.86% | 9.38% | 11.44% | 12.32% | 10.90% | 32.85% | 21.06% | 14.22% | 10.33% | 13.35% | 10.64% | 17.08% | -6.18% |
| C | 8.10% | 10.25% | 9.16% | 11.65% | 8.40% | 9.00% | 9.43% | 13.18% | 19.95% | 13.10% | 8.64% | 8.10% | 12.43% | 12.57% | -3.14% |
| D | 9.90% | 10.20% | 8.30% | 10.40% | 9.62% | 8.10% | 9.42% | 15.10% | 7.40% | 9.90% | 8.60% | 8.10% | 13.40% | 10.42% | -1.00% |

**Table S7. The** **fattening days to reach 115kg**

| Farm | Fattening days before outbreak (6 months) | | | | | | | Fattening days after outbreak (10 months) | | | | | | | | | | | Difference |
| --- | --- | --- | --- | --- | --- | --- | --- | --- | --- | --- | --- | --- | --- | --- | --- | --- | --- | --- | --- |
|  | -6 | -5 | -4 | -3 | -2 | -1 | mean | +1 | +2 | +3 | +4 | +5 | +6 | +7 | +8 | +9 | +10 | mean |  |
| A | 176.20 | 176.86 | 174.70 | 174.52 | 180.57 | 179.59 | 177.34 | 179.03 | 174.42 | 175.21 | 177.64 | 176.31 | 173.70 | 182.52 | 179.54 | 179.59 | 177.54 | 178.58 | 1.23 |
| B | 176.89 | 172.45 | 174.97 | 170.56 | 173.30 | 177.85 | 173.54 | 172.63 | 174.32 | 172.93 | 176.29 | 173.57 | 171.59 | 178.26 | 196.33 | 195.24 | 182.84 | 183.59 | 10.05 |
| C | 177.62 | 179.70 | 181.11 | 180.35 | 178.23 | 180.00 | 179.92 | 182.79 | 180.01 | 178.29 | 175.15 | 177.91 | 186.84 | 195.34 | 190.15 | 177.61 | 179.83 | 185.95 | 6.03 |
| D | 169.26 | 172.67 | 170.43 | 172.31 | 174.98 | 169.35 | 171.50 | 168.42 | 170.44 | 171.69 | 170.35 | 170.72 | 169.98 | 173.06 | 178.87 | 181.00 | 183.69 | 177.32 | 5.82 |

**Table S8. The health costs (vaccination and medication costs,** ¥**)**

| Farm | Health costs before outbreak (6 months) | | | | | | | Health costs after outbreak (10 months) | | | | | | | | | | | Difference |
| --- | --- | --- | --- | --- | --- | --- | --- | --- | --- | --- | --- | --- | --- | --- | --- | --- | --- | --- | --- |
|  | -6 | -5 | -4 | -3 | -2 | -1 | mean | +1 | +2 | +3 | +4 | +5 | +6 | +7 | +8 | +9 | +10 | mean |  |
| A | 33.84 | 31.66 | 35.01 | 39.55 | 33.96 | 28.71 | 33.79 | 31.06 | 30.49 | 29.58 | 28.78 | 31.84 | 34.54 | 38.38 | 41.20 | 39.86 | 36.00 | 38.00 | 4.21 |
| B | 27.56 | 35.30 | 31.49 | 26.62 | 34.73 | 29.26 | 30.83 | 37.66 | 27.69 | 31.42 | 29.57 | 31.49 | 26.62 | 47.26 | 67.47 | 52.81 | 38.81 | 46.59 | 15.77 |
| C | 37.56 | 40.25 | 38.85 | 45.08 | 40.41 | 45.08 | 40.48 | 42.56 | 36.20 | 39.12 | 41.80 | 38.28 | 53.85 | 66.12 | 54.08 | 36.75 | 40.47 | 50.25 | 9.78 |
| D | 29.12 | 21.94 | 28.00 | 29.45 | 37.34 | 34.33 | 30.03 | 35.00 | 32.95 | 28.96 | 27.84 | 31.50 | 34.21 | 42.17 | 37.00 | 51.50 | 44.41 | 41.86 | 11.83 |
